# Pembrolizumab-induced nephrotoxicity in a patient with breast cancer

**DOI:** 10.1177/17588359241248362

**Published:** 2024-04-25

**Authors:** Samer Alkassis, Kasey Fitzsimmons, Sara Hurvitz

**Affiliations:** Division of Hematology/Oncology, University of California Los Angeles, Los Angeles, CA, USA; Division of Hematology/Oncology, UCLA Santa Monica Parkside, Santa Monica, CA, USA; Division of Hematology/Oncology, Fred Hutchinson Cancer Center, University of Washington School of Medicine, Seattle, WA, USA

**Keywords:** adverse event management, breast cancer, checkpoint inhibitor, immune-related adverse events < immunotherapy, immunotherapy

## Abstract

The introduction of immunotherapy has revolutionized the treatment and improved outcomes of multiple types of cancer. Although breast cancer is a less immune-responsive tumor type, the incorporation of pembrolizumab into chemotherapy regimens in the neoadjuvant and first-line metastatic setting for the triple-negative disease has improved outcomes. However, the use of this type of treatment is associated with a spectrum of adverse events. Although rarely affected, kidneys can be a target for immunotherapy, leading to irreversible injury if not recognized and addressed early. A 52-year-old woman presented with clinical stage II right breast cancer diagnosed at an outside facility. Neoadjuvant docetaxel/carboplatin/pembrolizumab every 3 weeks was started. Given the partial response on MRI after the 4th cycle, treatment was switched to doxorubicin/cyclophosphamide. However, pembrolizumab was held in cycle 2 due to the rash and then resumed in cycle 3 after the resolution of symptoms. Elevated creatinine was noted 3 weeks after the last dose of pembrolizumab without improvement despite adequate fluid resuscitation. Diagnostic workup was unremarkable except for pyuria and minimal albuminuria on urinalysis. In the absence of other risk factors and the temporal relationship between pembrolizumab administration and the onset of acute kidney injury (AKI), immune-related nephrotoxicity was the underlying diagnosis. After initiation of corticosteroids, creatinine decreased back to baseline without the need for kidney biopsy. An addendum to the original pathology report from the outside facility surfaced 5 months after starting treatment, revealing that the second breast lesion had a Fluorescence in situ hybridization (FISH) test performed that was positive. Given this fact, therapy was changed to two cycles of neoadjuvant paclitaxel/carboplatin/trastuzumab/pertuzumab, with approximately 8 weeks between the last pembrolizumab dose and the first dose of trastuzumab. Thereafter, she underwent a right breast mastectomy which showed residual invasive carcinoma with negative margins and lymph nodes. She completed 1 year of trastuzumab. Immune-related AKI is a rare, but potentially serious complication associated with an increase in mortality. Further research is needed in the development and early detection. There is promising research in the development of noninvasive biomarkers which has the added benefit of identifying patients who can be re-challenged with immunotherapy.

## Introduction

The introduction of immunotherapy has revolutionized the treatment and improved outcomes of multiple types of cancer. Although breast cancer is a less immune-responsive tumor type, the incorporation of the immune checkpoint inhibitor (ICI) pembrolizumab into chemotherapy regimens in the neoadjuvant and first-line metastatic setting for the triple-negative disease has improved outcomes.^1,2^ While these improvements have had minimal clinical impact, the use of pembrolizumab is associated with a spectrum of immune-related adverse events (irAE). Most commonly, these include dermatologic, gastrointestinal (GI), and endocrine dysfunction. Although rarely affected, kidneys can be a target for ICI, leading to irreversible injury if not recognized and addressed early. Herein, we present a case of immune-mediated renal toxicity in a patient treated with a combination of chemotherapy and pembrolizumab. The reporting of this case conforms to the CARE guidelines (Supplemental Material 1).

## Case

**Figure 1.**
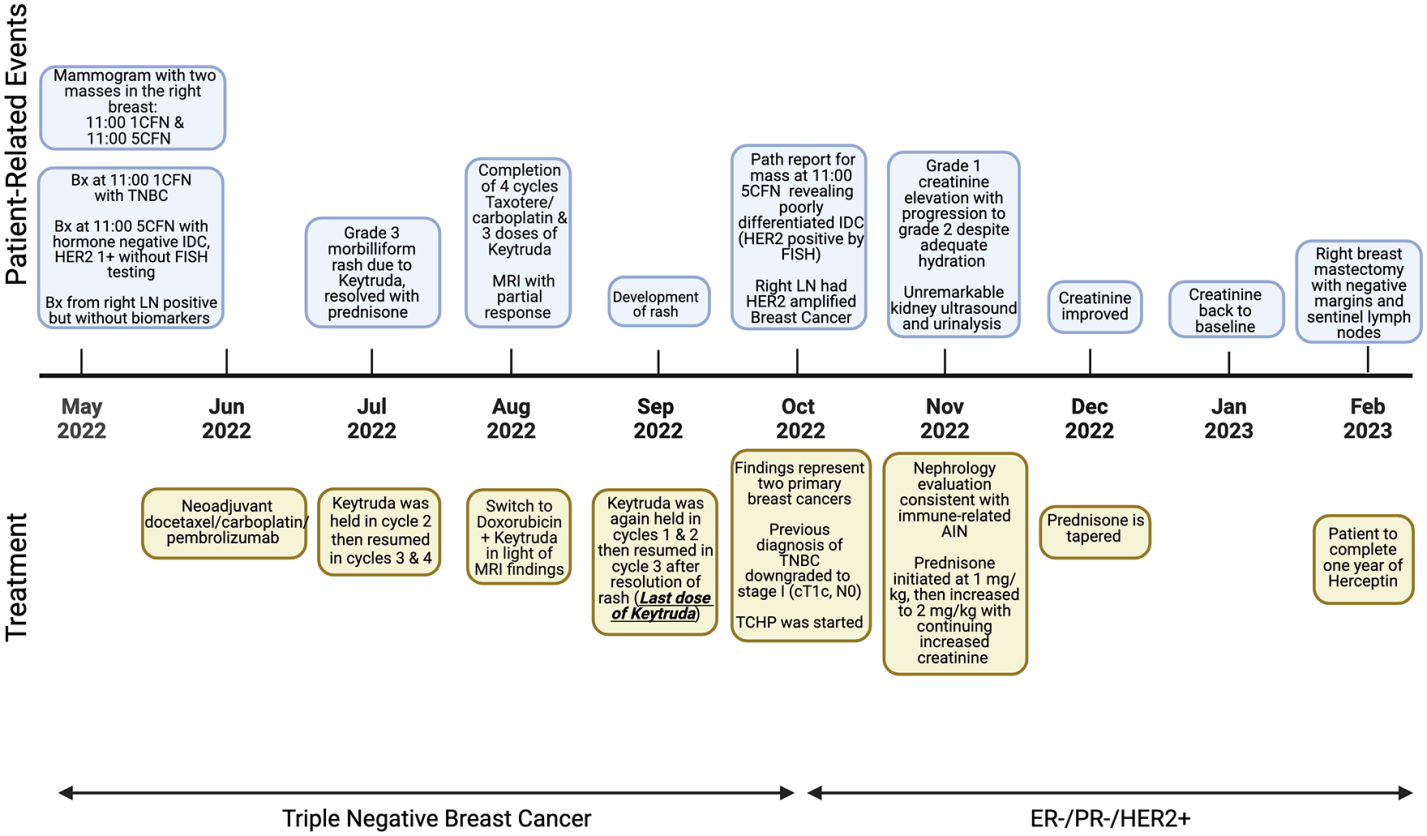
Patient’s timeline of events. Source: BioRender.com (2024). Bx, biopsy; CFN, cm from the nipple; IDC, invasive ductal carcinoma; LN, lymph node; TCHP, taxotere/carboplatin/herceptin, pertuzumab; TNBC, triple-negative breast cancer.

A 52-year-old woman presented to the clinic with clinical stage II (cT2cN1) triple-negative right breast cancer diagnosed at an outside facility (Figure 1). The initial mammogram showed several upper outer quadrant lesions (largest 1.1 × 0.8 cm) and multiple enlarged axillary lymph nodes. Biopsy of the first lesion in the breast (at 11 O’clock, 1 cm from the nipple) revealed poorly differentiated invasive ductal carcinoma (IDC), estrogen (ER)/progesterone (PR) negative, HER2 1+ immunohistochemical (IHC) and negative by FISH (ratio 1.4, copy number 3.4) with a Ki67 of 60%. Biopsy of a second lesion (at 11 O’clock, 5 cm from the nipple) revealed poorly differentiated IDC, estrogen ER/PR negative, HER2 1+ IHC (FISH not initially performed) with Ki67 53%. A biopsy of a right axillary lymph node was positive for metastasis without FISH or biomarker testing. Staging scans did not show evidence of distant metastases. Neoadjuvant docetaxel/carboplatin/pembrolizumab every 3 weeks was started. Pembrolizumab was held in the second cycle due to Grade 3 rash requiring high-dose corticosteroid, then resumed on cycles 3 and 4.

A repeat MRI after the 4th cycle was obtained to assess response to neoadjuvant therapy, per the surgeon’s request, as there were areas of concern in the right breast and abnormal lymph nodes not appreciated on prior imaging. This showed a partial response (from 3.8 × 3.5 cm in conglomerate to 3 × 3 cm). Doxorubicin/cyclophosphamide was initiated; however, pembrolizumab was again held on cycles 1 and 2 due to recurrent rash and then resumed on cycle 3 after resolution of rash.

Elevated creatinine from baseline 0.7 (Glomerular filtration rate (GFR) >89 mL/min/1.73 m^2^) to 1.4 (GFR 44 mL/min/1.73 m^2^) was noted 3 weeks after the last dose of pembrolizumab, which was highly suspicious for ICI-induced AKI per nephrology evaluation. She was not on Nonsteroidal anti-inflammatory drugs (NSAID) but on pantoprazole. Renal ultrasound showed normal kidneys without hydronephrosis. Urinalysis showed sterile pyuria and minimal albuminuria. Despite adequate hydration, her kidney function continued to worsen (GFR 28 mL/min/1.73 m^2^). Given this fact, and in the absence of other identified etiologies, immune-mediated nephrotoxicity was the underlying diagnosis. It was felt that waiting for the kidney biopsy without intervening would risk further deterioration of the kidney function. After having a balanced discussion with the patient, we jointly decided to proceed with treatment for ICI-related AKI. She started on prednisone 1 mg/kg which was increased to 2 mg/kg with rapid improvement in kidney function. Pantoprazole was switched to famotidine as it could potentiate immune-related acute interstitial nephritis (AIN), and corticosteroids were tapered after normalization of creatinine.

At this time, an addendum to the original pathology report from the outside facility surfaced, revealing that the second breast lesion (at 11 O’clock, 5 cm from the nipple) had a FISH test performed (after the original finalized pathology report) that was positive (ratio 2.9, copy 8.3). Our institution was not notified of this significant update until 5 months after starting treatment. Pathology of both breast lesions and lymph node biopsy was repeated at the University of Sothern California and it was confirmed patient had triple-negative breast cancer (TNBC) in the first breast lesion (at 11 O’clock, 1 cm from the nipple), an ER/PR-, HER2+ breast cancer in the second lesion (at 11 O’clock, 5 cm from the nipple), and the lymph node had HER2 amplified breast cancer. Given the fact that the lymph node metastasis was from the HER2+ tumor, not TNBC, it became clear that the TNBC was stage I. That said, therapy was changed to two cycles of neoadjuvant paclitaxel/carboplatin/trastuzumab/pertuzumab (TCHP), with approximately 8 weeks between the last pembrolizumab dose and the first dose of trastuzumab. TCHP would have been initiated from the beginning if we knew TNBC was stage I and HER2+ tumor was stage II as TC would treat TNBC. Pembrolizumab was not resumed as the use of immunotherapy in stage I TNBC is not standard of care, and due to the history of nephrotoxicity. She received three doses of weekly paclitaxel, one dose of carboplatin, and two doses of trastuzumab/pertuzumab. Thereafter, she underwent a right breast mastectomy which showed 1.5 mm of residual tumor from TNBC lesion with negative margins. There were no metastases in the three sentinel lymph nodes removed. There was a completed pathologic response in the HER2+ tumor after neoadjuvant therapy. Given this fact, trastuzumab emtansine was not indicated, and the patient completed 1 year of trastuzumab. She has fully recovered without evidence of disease.

## Discussion

Immune-related toxicities, a spectrum of autoimmune events, are side effects uniquely associated with ICI. From most to least common; dermatologic, GI, hepatic, endocrine, and pulmonary toxicities have been reported in the literature.^3^ Interestingly, the kidney is a rare target for this type of therapy with ICI-induced AKI reported in 2.2–5% of patients in prior retrospective trials.^4^ In breast cancer, immune-related AKI has been reported in <1% of ICI prospective trials (Table 1). Kidney injury can happen at any point during immunotherapy, ranging from a few days after the introduction of the medication to >10 weeks after the end of treatment.^3^

**Table 1.** Incidence of irAKI in breast cancer ICI phase III trials.

Trial	irAKI incidence (%)	Breast cancer subtype
Pembrolizumab		
Keynote 119^5^	<1	Metastatic TNBC
Keynote 522^2^	0	Early-stage TNBC
Keynote 355^1^	0	Advanced TNBC
Atezolizumab		
Impassion 131^6^	0.5	Unresectable locally advanced/metastatic TNBC
Impassion 130^7^	0	Advanced TNBC
Impassion 050^8^	0	Early-stage HER2-positive breast cancer
Impassion 031^9^	0	Early-stage TNBC
NeoTRIPaPDL1^10^	<1	Early, high-risk/advanced TNBC

ICI, immune checkpoint inhibitor; irAKI, immune-related acute kidney injury; TNBC, triple-negative breast cancer.

### Pathophysiology

**Figure 2.**
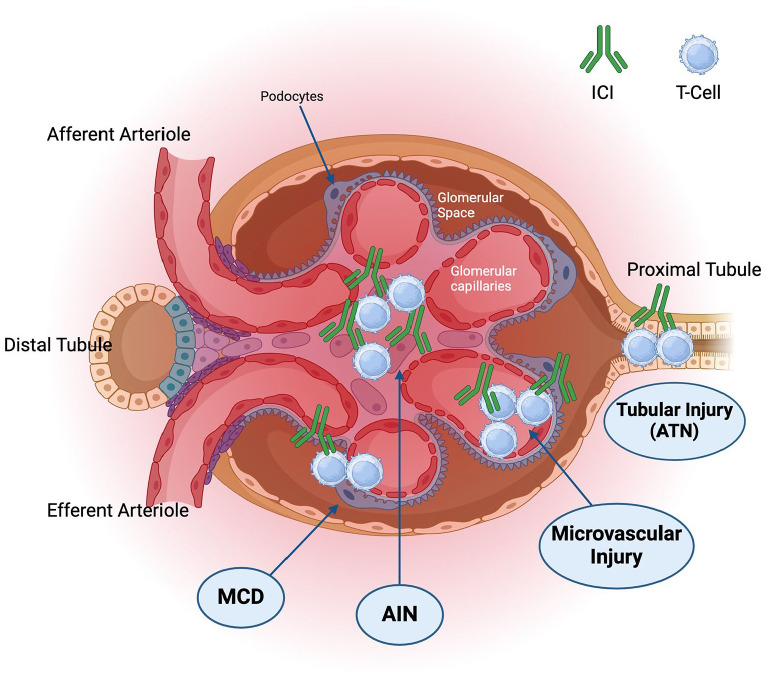
Pathophysiology of immune-related acute kidney injury. Source: ‘Glomerulonephritis’, by BioRender.com (2024). AIN, acute interstitial nephritis; ATN, acute tubular necrosis; ICI, immune checkpoint inhibitor; MCD, minimal changes disease.

AIN is the predominant pathophysiology associated with ICI-related nephrotoxicity. However, tubular, glomerular, and microvascular injury have also been reported leading to acute tubular necrosis (ATN), microangiopathic thrombosis, and glomerular injury, respectively (Figure 2).^3^ In a large case series reported by Izzedine *et al.*^11^ pembrolizumab-related AKI incidence was 1.77% (12 out of 676 patients). AIN and ATN were the most common findings on pathology, whereas minimal changes in disease occurred in two patients.

### Pembrolizumab and breast cancer

In 2021, the FDA approved the use of pembrolizumab in combination with chemotherapy for the neoadjuvant treatment of early-stage triple-negative breast cancer, based on the KEYNOTE-522 trial which showed a significant increase in the proportion of patients achieving pathologic complete response when compared to placebo + chemotherapy alone.^2^ There was an increased incidence of irAEs as well as an increased incidence of higher grade irAEs in the pembrolizumab group, compared to placebo (43% *versus* 22%, 15% *versus* 2%, respectively).^12^ In the setting of early-stage TNBC, which is largely curative with standard chemotherapy, the added toxicity of pembrolizumab, which included four fatalities directly related to irAEs, was considered carefully by the FDA, which at first denied accelerated approval of pembrolizumab until more survival data were available. During the initial pembrolizumab trials,^12^ renal insufficiency was very rarely reported. In fact, in the Schmid *et al*.’s^13^ trial for locally advanced or metastatic TNBC, zero instances of renal dysfunction were reported, and in the phase III trial of pembrolizumab for melanoma, only two patients were observed to have immune-related AKI (irAKI).^14^ However, with the subsequent approval of pembrolizumab, as well as other ICIs now entering clinical trial testing, more ICI-related renal toxicities are being reported, necessitating a greater focus on this significant and potentially irreversible side effect. In addition, since steroids are the mainstay treatment for most irAEs, under-recognition of ICI-related AKI may occur as steroids are used for other non-renal complications such as skin or GI reactions. Nevertheless, ICI-induced nephrotoxicity remains a potentially serious complication that needs to be screened and monitored carefully in this population.

### Predisposing factors for irAKI development

Overall, the incidence of irAKI is relatively uncommon; however, recognizing early renal involvement, or better yet identifying patients with high risk through prescreening, is critically important to prevent irreversible kidney damage. Several proposed demographics may be associated with an increased risk of irAKI, including prior autoimmune condition, female gender, baseline low GFR, dual ICI therapy, and proton pump inhibitor (PPI) use. Although a history of autoimmune disease has been a contraindication to the use of ICI, in practice many patients with autoimmune diseases (psoriasis, scleroderma, Graves’ disease) do receive this therapy when the treatment benefit outweighs the risk. Several studies^15,16^ have shown that these patients obtain similar clinical benefits compared to a standard population; however, the rate of AKI-specific irAEs for these patients is increased compared to counterparts without a prior history of autoimmune disease. One study involving 1766 patients who received ICI revealed that 14% of patients with a known history of auto-immune disease [odds ratio (OR) = 5.88, *p* = 0.04] and 57% of patients with prior immune-associated toxicity (OR = 1.31, *p* = 0.01) developed irAKI, and thus these prior conditions are significant predictors of ir-AKI.^17^ While further research is needed to ascertain whether this represents the progression of a pre-existing autoimmune disease *versus* a true ICI-related adverse event, it stands that there remains an increased risk for this population who should be monitored closely for the development of AKI.

In a series reported by Cortazar *et al*.,^18^ 138 patients who had irAKI were screened for pre-treatment risk factors, 3 of which were identified as being significantly related to the development of irAKI; low baseline eGFR (<60 mL/min/1.73 m^2^), dual ICI therapy, and concomitant use of PPI. It is notable that ICIs are not cleared renally but are primarily cleaved by lysosomal proteolytic degradation.^19^ For that reason, ICIs do not need dose adjustment for kidney impairment and have been safely managed in end-stage renal disease.^20^ However, patients with advanced CKD have shown an increased risk of ICI-induced AKI; therefore, it is highly recommended that careful evaluation of renal function should be done before and during treatment.

Concomitant administration of other drugs may also increase the risk of irAKI. It is well established that PPIs increase the risk of AKI in the general population, raising a valid question: how can providers distinguish between an ICI-related AKI from other drug-induced AKIs and is drug administration in combination contraindicated? Several murine-based melanoma research models have shown promising results with the use of dual checkpoint inhibitors, increasing T-cell numbers more than monotherapy ICI. Wolchok *et al.*^21^ applied this approach to melanoma patients with ipilimumab and nivolumab, and while it had a promising clinical response, it was associated with developing grade 3 and grade 4 toxicities, 6% of which involved the kidneys. Extra caution should be given to patients treated with more than one ICI given that they not only have a higher risk of developing an irAKI but also a higher grade of toxicity. Although in breast cancer the use of dual ICI-based therapy is not standard practice, the use of antibody-based therapy that may stimulate antibody-dependent cellular cytotoxicity is standard practice in HER2-positive breast cancer. Our patient’s presentation was consistent with two primary breast cancers with different biomarkers: one that was HER2 amplified and one that was triple negative. Thus, treatment with an ICI and a HER2-targeted therapy sequentially was indicated. It is worth mentioning that this patient did not receive trastuzumab and pembrolizumab at the same time; however, the long half-life of antibody therapy (26 days for pembrolizumab) may have led to their co-presence for a period of time. The combination of ICI with trastuzumab-based therapy has been well tolerated.^22,23^ If our patient did not have a serious irAE compromising her kidney function and had stage II TNBC, we would have continued immunotherapy and trastuzumab.

### Drug-related AKI *versus* irAKI?

There are currently no reliable differentiating features that distinguish ICI-related AKI from AKI due to other drugs. Therefore, drugs known to cause nephrotoxicity, such as PPIs, should be used with caution in patients receiving ICI therapy. In addition to drug-related kidney damage, there are several other common causes of AKI in cancer treatment such as volume depletion and sepsis. Distinguishing these various etiologies of AKI remains quite complex, and the current gold standard diagnosis involves a renal biopsy, which remains an invasive and time-consuming procedure. One common theme with ICI-related AKI is the significant latency between the start of treatment and the development of renal dysfunction, ranging from several weeks to months, with the median time being approximately 14 weeks.^24^ That said, a prolonged timeline alone is not enough to differentiate ICI-related AKI from other drug-induced causes. While the classic triad of rash, fever, and eosinophilia within days of drug initiation is well established for the most frequent offenders of drug-induced AIN (antibiotics, NSAIDS, and PPIs), less than 10% of patients present this way, with most having a slow, indolent course of progressive kidney function decline in much the same timeframe as ICI-related AKI.^25^ It has been hypothesized that this delayed onset of AKI has to do more with the prolonged longevity of activated T cells rather than the direct toxic effects of the ICIs themselves, mimicking an autoimmune process than a true drug hypersensitivity.^26^ Hence, the relationship between irAKI and subsequent morbidity/mortality is difficult to distinguish, as biopsy-proven irAKI is the only current reliable way to distinguish true irAKI *versus* other etiologies. Furthermore, biopsies tend to be performed rarely, as management with corticosteroids remains the mainstay of treatment with little influence from invasive biopsy results. A study by Baker *et al.*^27^ with the largest cohort of irAKI to date of this publication concluded that nonspecific AKI was independently associated with lower survival of patients treated with ICI, particularly in the first 120 days of its occurrence. However, the subgroup of patients with biopsy-proven irAKI, which tends to present weeks to months after immunotherapy exposure, had higher survival than those without irAKI, indicating that this IrAE may be a marker of therapeutic response to ICI. This result further highlights the effectiveness of ICI despite the small risk of irAKI, thus accurate and expedient identification of irAKI continues to represent a priority to clinicians who utilize this promising immunotherapy.

While promising research is underway for the development of biomarkers to accurately diagnose ICI-induced AKI, there currently remains no clear way to differentiate irAKI from drug-induced AKI. Current practice, outside of the gold standard of renal biopsy, is based on predictive, not definitive, test results, such as high neutrophil–lymphocyte ratio and increased eosinophil/monocyte count. There is new but limited evidence that other biomarkers (cytokines, autoantibodies, Human leukocyte antigen (HLA) genotypes, microRNA, and gene expression profiling) may provide a fast and accurate assessment of irAKI. Ongoing research, including prospective disease and agent-specific studies, is still required to provide critical insight into this aspect of ICI therapy adverse effect management. These noninvasive biomarkers may have the ability to identify patients in the early stages of irAKI, before potentially irreversible kidney damages occur, and have the added utility of monitoring patients who undergo re-challenging with ICI after an episode of irAKI, conserving this effective avenue of treatment. With ongoing fast-expanding approvals and trials for ICIs in both breast cancer and other organ systems, these noninvasive biomarkers represent a solution to a current lack of diagnostic ability in ICI-related oncologic care.

### Management of irAKI

Given the rarity of this toxicity, multidisciplinary management is recommended. The American Society of Clinical Oncology and the National Comprehensive Cancer Network have developed guidelines for immune-related nephrotoxicity treatment.^28^ For persistent grade 2 (doubling of creatinine or higher), ICI should be discontinued in addition to corticosteroid administration (0.5–1 mg/kg/day of prednisone for grade 2 and 1–2 mg/kg/day for grade 3). Once creatinine decreases to grade 1 (creatinine no more than 1.5 times above baseline), a taper can be initiated over 4–6 weeks.

Although treatment-related toxicity poses a significant challenge in therapy continuation, the association with improved clinical outcomes and survival has been observed.^29–31^ In a retrospective analysis conducted by Fujii *et al.*,^29^ patients with a variety of tumor types (breast, colorectal, pancreatic, hepatocellular carcinoma (HCC), renal cell carcinoma (RCC), head & neck (H&N), melanoma, non-small cell lung cancer (NSCLC)) who developed irAE were re-challenged with ICI after treatment with corticosteroids and four out of five demonstrated continuous response. Interestingly, the overall response was higher and the median time to progression was longer in patients with grade ⩾3 toxicity. In another study, irAE was more frequently reported in patients with clinical response to PD-1/PD-L1 antibodies, and the duration of response was not affected by glucocorticoid treatment.^30^

## Conclusion

ICIs are an important treatment option in TNBC,^1,2^ with a phase III trial showing both a significant improvement in pathological complete response rate as well as invasive disease-free survival when added to standard neoadjuvant chemotherapy.^2^ IrAKI is a rare, but potentially serious complication associated with an increase in mortality. Medical oncologists have the unique role of being the leaders of a multidisciplinary team in the treatment of breast cancer, and when driving the management of patients with this disease, early recognition, and prompt intervention of irAEs remain critical to maintain effective response to treatment and patient safety. Renal biopsy remains the gold standard for diagnosis; however, it is an invasive and non-expedient procedure that is often performed after potentially irreversible kidney damage has already occurred. Further research is needed in the development and early detection of irAKI, as there remains a lack of sensitive or specific clinical features for reliable early diagnosis, and a high index of clinician suspicion is still needed.^32^ There is promising research in the development of noninvasive biomarkers (e.g. urinary, blood, and imaging-based biomarkers), which has the added benefit of identifying patients who can be re-challenged with ICI after an episode of ICI-AKI *versus* foregoing that avenue of treatment. Additional validation studies are needed to fully characterize their clinical usefulness in combination with other markers.

## Supplemental Material

sj-pdf-1-tam-10.1177_17588359241248362 – Supplemental material for Pembrolizumab-induced nephrotoxicity in a patient with breast cancer Supplemental material, sj-pdf-1-tam-10.1177_17588359241248362 for Pembrolizumab-induced nephrotoxicity in a patient with breast cancer by Samer Alkassis, Kasey Fitzsimmons and Sara Hurvitz in Therapeutic Advances in Medical Oncology

## Declarations

**Disclaimer:** Sara Hurvitz is an associate editor of *Therapeutic Advances in Medical Oncology* and an author of this paper; therefore, the peer-review process was managed by alternative members of the board and the submitting editor has not been involved in the decision-making process.

**Ethics approval and consent to participate:** Not applicable.

**Consent for publication:** The patient provided written informed consent for publication of this case report, including any accompanying data or images.

**Competing interests:** S. A.: None; K. F.: None; S. H.: Contracts and grants (paid to institutions for clinical trial-related work): Ambrx, Amgen, Arvinas, Astra Zeneca, Bayer, Celcuity, Cytomx, Daiichi-Sankyo, Dantari, Dignitana, Genentech/Roche, G1-Therapeutics, Gilead, Greenwich Life Sciences Inc., GSK, Immunomedics, Eli Lilly, LOXO, Macrogenics, Novartis, OBI Pharma, Orinove, Orum, Pfizer, Phoenix Molecular Designs, Ltd., Pieris, PUMA, Radius, Samumed, Sanofi, Seattle Genetics/Seagen, Zymeworks. Data Safety Monitoring Board: Alliance Foundation, QuantumLeap (I-SPY) and InClin/Atossa. Spouse: ROMTech (stocks).

**Availability of data and materials:** Not applicable.
