# Supplementary material for: Pembrolizumab-induced nephrotoxicity in a patient with breast cancer
Source: Ther Adv Med Oncol. 2024 Apr 25;16:17588359241248362. doi: 10.1177/17588359241248362 (PMC11047248; doi:10.1177/17588359241248362)
Supplement: sj-pdf-1-tam-10.1177_17588359241248362 – Supplemental material for Pembrolizumab-induced nephrotoxicity in a patient with breast cancer [file sj-pdf-1-tam-10.1177_17588359241248362.pdf]

CARE case report guidelines

CARE Checklist of information to include when writing a case report

(cc) BY-NC-ND

Topic	Item	Checklist item description	Reported on Line
Title	1	The diagnosis or intervention of primary focus followed by the words “case report”	
Key Words	2	2 to 5 key words that identify diagnoses or interventions in this case report, including "case report"	x
Abstract (no references)	3a	Introduction: What is unique about this case and what does it add to the scientific literature?	x
	3b	Main symptoms and/or important clinical findings	x
	3c	The main diagnoses, therapeutic interventions, and outcomes	x
	3d	Conclusion—What is the main “take-away” lesson(s) from this case?	x
Introduction	4	One or two paragraphs summarizing why this case is unique (**may include references**)	x
Patient Information	5a	De-identified patient specific information.	x
	5b	Primary concerns and symptoms of the patient.	x
	5c	Medical, family, and psycho-social history including relevant genetic information	x
	5d	Relevant past interventions with outcomes	x
Clinical Findings	6	Describe significant physical examination (PE) and important clinical findings.	x
Timeline	7	Historical and current information from this episode of care organized as a timeline	x
Diagnostic Assessment	8a	Diagnostic testing (such as PE, laboratory testing, imaging, surveys).	x
	8b	Diagnostic challenges (such as access to testing, financial, or cultural)	
	8c	Diagnosis (including other diagnoses considered)	x
	8d	Prognosis (such as staging in oncology) where applicable	x
Therapeutic Intervention	9a	Types of therapeutic intervention (such as pharmacologic, surgical, preventive, self-care)	x
	9b	Administration of therapeutic intervention (such as dosage, strength, duration)	x
	9c	Changes in therapeutic intervention (with rationale)	x
Follow-up and Outcomes	10a	Clinician and patient-assessed outcomes (if available)	x
	10b	Important follow-up diagnostic and other test results	x
	10c	Intervention adherence and tolerability (How was this assessed?)	x
	10d	Adverse and unanticipated events	
Discussion	11a	A scientific discussion of the strengths AND limitations associated with this case report	x
	11b	Discussion of the relevant medical literature **with references**.	x
	11c	The scientific rationale for any conclusions (including assessment of possible causes)	x
	11d	The primary “take-away” lessons of this case report (without references) in a one paragraph conclusion	x
Patient Perspective	12	The patient should share their perspective in one to two paragraphs on the treatment(s) they received	
Informed Consent	13	Did the patient give informed consent? Please provide if requested	Yes ☒ No ☐
